# A variant in SIRT2 gene 3′-UTR is associated with susceptibility to colorectal cancer

**DOI:** 10.18632/oncotarget.17460

**Published:** 2017-04-27

**Authors:** Yong Yang, Jie Ding, Zhi-Gang Gao, Zhen-Jun Wang

**Affiliations:** ^1^ Department of General Surgery, Beijing Chao-Yang Hospital, Capital Medical University, Beijing 100020, China

**Keywords:** SIRT2, polymorphism, colorectal cancer, risk

## Abstract

SIRT2 is a member of sirtuin family and is associated with cell growth in various cancers. In this study, we searched for variants in functional region of *SIRT2* gene and identify rs2015 and rs2241703 in the 3′UTR with minor allele frequency >0.05 in Chinese Han Beijing population from 1000 Genomes Project. We then genotyped these two variants in 842 colorectal cancer (CRC) patients and 1,718 healthy controls using Taqman genotyping assay. Association between variants and risk of CRC is calculated using logistic regression adjusted for sex and age. We found that rs2015C was significantly associated with increased risk of CRC. Compared with CC genotype carriers, CA genotype and AA genotype carriers were associated with CRC susceptibility with OR being 0.79 (95% CI: 0.65–0.96, *P* = 0.019) and 0.73 (95% CI: 0.58–0.92, *P* = 0.009), respectively. When stratified by sex and age, significant associations were observed only in males (OR = 0.82, 95% CI: 0.71–0.96, *P* = 0.010) for rs2015, but not females (OR = 0.90, 95% CI: 0.73–1.10, *P* = 0.287). It is suggested that the sequence including rs2015C allele lies within a binding site for the full seed region of hsa-miR-376a-5p. Through a systematic interrogate of variants in the functional region of *SIRT2* gene, we identified rs2015 was significantly associated with CRC susceptibility, providing new insights into the carcinogenesis of CRC.

## INTRODUCTION

Colorectal cancer (CRC) ranks the third leading cause of cancer in males and the second in females [1]. CRC incidence rates increased from 1975 through the mid-1980s in both men and women but have since decreased with the exception of a slight, unexplained interruption from 1996 to 1998 in the United States [2]. However, with the progressive ‘westernization’ of lifestyles and increasing obesity, the incidence rates of CRC is sharply increasing in East Asian countries, including China [3]. In China, the age-standardized CRC incidence rates per 100,000 persons were 215.7 in men and 160.6 in women in 2015. The increased CRC incidence could be due to lifestyles and obesity, though these are insufficient to explain the high incidence in China. Several environmental factors have been established to play a vital role in the etiology of CRC, it is reported that about 35% of CRC risk may be attributable to genetic factors [4]. Several genome-wide association study (GWAS) have identified approximately 20 genetic loci in genes and long non-coding RNAs in Asian associated with CRC susceptibility [5–8], these could only explain a small part of the heritability. Therefore, more variants, especially functional variants in susceptibility gene of CRC still need to be further explored.

Silent information regulator 2 (SIRT2), encoding a member of the sirtuin family of proteins (SIRT1–SIRT7), belongs to a highly conserved family of NAD+-dependent deacetylases. Sirtuin family have different tissue and subcellular localization. SIRT2 are expressed in the nucleus and cytoplasm. Several proteins, such as histones, α-tubulin, transcription factors and enzymes are the substrates of SIRT2 [9–11]. Experiments using knockout SIRT2 in mice showed that SIRT2 is a tumor suppressor through its role in regulating mitosis and genome integrity [12]. Studies also have showed that SIRT2 is involved in the tumorigenesis of many types of cancer, including non-small-cell lung cancer, glioma, melanoma and CRC.

Accumulating evidence has shown that single-nucleotide polymorphisms (SNP) located in the 3′-untranslated region (3′-UTR) of genes might affect gene regulation and are associated with individual susceptibility to cancer [13]. The aim of the present study was to identify important SNP in the functional region (including promoter, 5′-UTR, coding region and 3′-UTR) of *SIRT2* gene which might be associated with CRC risk. We searched for SNPs with minor allele frequency > 0.05 in Chinese Han Beijing population from 1000 Genomes Project in *SIRT2* in the functional region and then genotyped SNPs in a case-control study to test whether these SNPs is associated with CRC risk.

## RESULTS

### Characteristic of study subjects

The distributions of demographic characteristics including sex and age of the CRC patients and controls involved in this study were showed in Table 1. 842 CRC patients and 1,718 gender matched healthy controls were used in the analysis. There were 66.9% and 70.8% males in cases and controls, respectively. The average age of cases and controls were 60.67 ± 12.99 (mean ± S.D.) years and 62.77 ± 9.81 years, respectively.

**Table 1:** Summary of characteristics of study subjects

	Cases (*n* = 842)	Controls (*n* = 1,718)
Age (years), mean ± S.D.	60.67 ± 12.99	62.77 ± 9.81
Gender, *n* (%)		
Male	539 (66.9)	1,216 (70.8)
Female	303 (33.1)	502 (29.2)

### Association between SNPs in functional region of *SIRT2* genes and CRC risk

We found two variants located in the 3′-UTR of *SIRT2* meeting our criteria and genotype frequencies of these two SNPs and their associations with risk of CRC were showed in Table 2. rs2015 significantly associated with CRC susceptibility. Compared with CC genotype carriers, CA and AA genotype carriers were associated with risk of CRC with OR being 0.79 (95% CI: 0.65–0.96, *P* = 0.019) and 0.73 (95% CI: 0.58–0.92, *P* = 0.009), respectively. On the other hand, we did not observe significant associations between rs2241703 CRC susceptibility (GA vs. GG: OR = 0.88, 95% CI: 0.75–1.04, *P* = 0.135; AA vs. GG: OR = 0.85, 95% CI: 0.67–1.08, *P* = 0.189).

**Table 2:** Association between two SNPs in 3′-UTR of SIRT2 gene and CRC risk in a Chinese population

SNP	Chr	Position	Genotype	Cases No. (%)	Controls No. (%)	OR (95% CI)a	*P*^a^
rs2015	19	39369369	CC	252 (29.9)	427 (24.9)	1.00 (Reference)	
			CA	415 (49.3)	884 (51.5)	0.79 (0.65–0.96)	0.019
			AA	175 (20.8)	407 (23.7)	0.73 (0.58–0.92)	0.009
			CA+AA	590 (70.1)	1,291 (75.1)	0.77 (0.64–0.93)	0.006
rs2241703	19	39369514	GG	671 (79.7)	1,335 (77.7)	1.00 (Reference)	
			GA	162 (19.2)	364 (21.2)	0.89 (0.73–1.10)	0.294
			AA	9 (1.1)	19 (1.1)	1.02 (0.46–2.27)	0.964
			GA+AA	171 (20.3)	383 (22.3)	0.90 (0.73–1.10)	0.308

^a^Calculated by logistic regression with adjustment for sex and age.

### Stratified analysis of SNPs in *SIRT2* genes and CRC risk

We performed stratified analyses by age and sex to evaluate the effects of variant genotypes on the risk of CRC (additive model: A vs. C) (Table 3). Among the males, rs2015 significantly associated with CRC susceptibility with an odds ratio of 0.82 (95% CI: 0.71–0.96, *P* = 0.010). However, the association were not observed for females (OR = 0.90, 95% CI: 0.73–1.10, *P* = 0.287). When stratified by age, rs2015 were significantly associated with CRC risk in older (> 60 years) patients, but not in younger (≤ 60 years) patients, with the OR being 0.81 (95% CI: 0.69–0.95, *P* = 0.010) and 0.89 (95% CI: 0.75–1.07, *P* = 0.216), respectively. For rs2241703, no significant associations were observed in stratified analyses.

**Table 3:** Association of SNPs and risk of CRC stratified by sex and age (additive model, A vs. C)

SNP	All samples		Male		Female	
	OR (95% CI)	*P*	OR (95% CI)	*P*	OR (95% CI)	*P*
rs2015	0.85 (0.75–0.96)	0.007	0.82 (0.71–0.96)	0.010	0.90 (0.73–1.10)	0.287
rs2241703	0.92 (0.76–1.11)	0.385	0.91 (0.72–1.15)	0.418	0.92 (0.66–1.28)	0.623
	All samples		> 60 years		≤ 60 years	
	OR (95% CI)	*P*	OR (95% CI)	*P*	OR (95% CI)	*P*
rs2015	0.85 (0.75–0.96)	0.007	0.81 (0.69–0.95)	0.010	0.89 (0.75–1.07)	0.216
rs2241703	0.92 (0.76–1.11)	0.385	0.95 (0.74–1.21)	0.660	0.86 (0.63–1.14)	0.266

### Function of the SNP in the *3′UTR* of *SIRT2* gene

In silico analysis using MirSNP showed that the rs2015 lies within a binding site for the full seed region of hsa-miR-376a-5p. The C allele matches the predicted hsa-miR-376a-5p seed–binding domain, whereas the A allele represents a mismatch base pairing.

## DISCUSSION

In the past ten years, several GWASs have reported approximately 20 genetic loci in genes and long non-coding RNAs in Asian associated with CRC risk [5–8]. Despite the discovery of these susceptibility loci, the heritability of CRC remains poorly understood. In the present study, we explored the association between SNPs with minor allele frequency > 0.05 in Chinese Han Beijing population in the functional region of *SIRT2* gene and CRC susceptibility in 842 CRC patients and 1,718 controls. Only two SNPs (rs2015 and rs2241703) located in 3′-UTR of *SIRT2* gene met our criteria. We found the frequency of the CA/AA genotypes of the rs2015 were significantly lower in patients than that in controls. rs2015A was associated with lower CRC risk in males, however, that association were not found for females. rs2015 was also associated with CRC risk in older (> 60 years). In silico analysis showed that the rs2015C allele matches the predicted hsa-miR-376a-5p seed–binding domain. Published study also suggested that hsa-miR-376a-5p is expressed in tissue of CRC [14]. These results were consistent with the fact that C allele is the risk allele for CRC. The interaction between hsa-miR-376a-5p and rs2015C allele results in lower expression of SIRT2. All these results from our present study suggest that rs2015 polymorphism in *SIRT2* gene was significant associated with CRC risk, indicating an important role of SIRT2 in CRC carcinogenesis.

The *SIRT2* gene is located at chromosome 19q13.2, and has been identified in a various of cancers. SIRT2 acts as a tumor suppressor gene in human gliomas through the regulation of microtubule network [15]. SIRT2 suppresses non-small cell lung cancer growth by targeting JMJD2A [16]. SIRT2 inhibits non-small cell lung cancer cell growth through impairing Skp2-mediated p27 [17]. SIRT2 downregulation confers resistance to microtubule inhibitors by prolonging mitotic arrest [18]. Several studies about SIRT2 also have been reported. Inhibition of SIRT2 expression also confers resistance to targeted therapies in KRAS-mutant colon cancers [19]. Negative SIRT2 protein expression was found in primary tumor samples, though this protein expressed in samples from CRC metastasis. Inhibition of SIRT2 using AK-1 would be a beneficial intervention in the treatment of CRC. Positive SIRT2 expression is associated with the CRC progression, however, SIRT2 has an antitumor role in CRC [20, 21].

Considerable evidence has shown that SNPs located in the 3′-UTR of genes have roles in the diagnosis, treatment outcome and survival of patients with CRC. A SNP which could alter let-7 microRNA-binding site in the 3′-UTR of KRAS gene predicts response in wild-type KRAS patients with metastatic CRC treated with cetuximab monotherapy [22]. One functional variant (rs1044129) in the 3′-UTR of RYR3 may be a potential marker for prognosis in patients following curative surgery for CRC [23]. Genetic variants in 3′-UTRs of MTHFR predict CRC susceptibility in Koreans [24]. Although SIRT2 play an important role in the prognosis of cancer, no study has reported that SNPs is associated with cancer. However, only one SNP (rs10410544) located in intron region of SIRT2 might be associated with risk of Alzheimer's disease [25–28].

Our present study also had some limitations. Despite a relatively large sample size, we only genotyped SNPs having MAFs ≥ 5% in the Han Chinese individuals from Beijing. Considering the statistical power of the sample size, some low-frequency SNPs might have got missed owing to the criteria used (CHB MAFs ≥ 5%). Second, the present case-control study only conducted in one center. More replication studies should be performed to confirm the association between rs2015 and risk of CRC. Third, the present only reported rs2015 is associated with risk of CRC. Functional experiments should be conducted to illustrate the mechanism for the association between the rs2015 in SIRT2 and susceptibility to CRC and validate them in CRC tissue sample.

In conclusion, through screen SNPs in the functional region of *SIRT2* gene, our study showed that rs2015 in the 3′-UTR of the *SIRT2* gene is associated with susceptibility to CRC. Compared to rs2015C allele, subjects with rs2015A was associated with lower CRC risk in males and older patients with age greater than 60. Further studies should be focus on the validation of this association in larger sample size and explore the mechanism of the function of this SNP.

## MATERIALS AND METHODS

### Study subjects

This study consists of 842 CRC patients and 1,718 controls. CRC patients were enrolled from Beijing Chao-Yang Hospital, Capital Medical University, Beijing, China between 2013 to 2016. Controls samples were selected from a community cancer screening program for early detection conducted in the same region during the same period as cases were collected. All CRC patients were confirmed primary CRC, without any radiotherapy or chemotherapy treatment prior to blood samples collected. Both cases and controls were Han Chinese descent. The informed consent was obtained from every participant at recruitment and peripheral blood samples and demographic characteristics such as gender, age and ethnicity were collected by interviewers. This study was conducted under the approval of the institutional review boards of Beijing Chao-Yang Hospital, Capital Medical University.

### SNP selection and genotyping

We searched for variants in the function region including promoter, 5′-UTR, coding region and 3′UTR of *SIRT2* gene with Chinese Han Beijing (CHB) minor allele frequency (MAF)>0.05. Two polymorphisms (rs2015 C>A and rs2241703 G>A) in the 3′-UTR of *SIRT2* gene were selected for further genotyping. Genomic DNA were extracted from 2 ml peripheral blood sample collected from each participant at recruitment. Genotyping were performed using Taqman SNP Genotyping method. The PCR primers and probes for genotyping rs2015 and rs2241703 were 5′- GGC CTG TGG CTA AGT AAA CCA TAC-3′/5′- GGC CCA CCA CCC ACT TTG-3′, 5′-FAM- CCT CTG AAT ATA ACC CAC AC-MGBNFQ-3′/5′-HEX- CCT CTG AAT CTA ACC CAC A-MGBNFQ-3′ and 5′- GGT TTA CTT AGC CAC AGG CCC-3′/5′- AGG CAT CTC TAC CAG CCC C-3′, 5′-FAM- CGT GGG GAC AGT TA-MGBNFQ-3′/5′-HEX- CGT GGG GGC AGT TA-MGBNFQ-3′. Several genotyping quality controls were implemented, including (i) the case and control samples were mixed in the plates, and persons who performed the genotyping assay were not aware of case or control status, (ii) positive and negative (no DNA) samples were included on every 384 well assay plate, and (iii) we further employed the direct sequencing of PCR products to replicate sets of 50 randomly selected, TaqMan genotyped samples for the two SNPs and the accordance between the two methods was 100%.

### miRNA biding site prediction

The MirSNP (bioinfo.bjmu.edu.cn/mirsnp/search/) were used to predict miRNA binding difference between two alleles of SNP. This tools were based on 7-nt seed alignment with all SNPs from dbSNP135. The analysis involved 1,921 different miRNAs.

### Statistical analysis

Unconditional multivariate logistic regression analysis were performed to assess the association. Odds ratios (ORs) and 95% confidence intervals (CIs) were calculated adjusted for age and sex. All statistical analyses were performed using SPSS software. All tests were two-sided and *P* < 0.05 were considered significant.
